# Effect of Medicaid Audio‐Only Telehealth Coverage Policy on Mental Health Visits in Federally Qualified Health Centers

**DOI:** 10.1111/1475-6773.70107

**Published:** 2026-03-26

**Authors:** Khyathi Gadag, Kanika Arora, Brian Kaskie, Whitney E. Zahnd

**Affiliations:** ^1^ Department of Social and Public Health College of Health Sciences and Professions, Ohio University Athens Ohio USA; ^2^ Department of Health Management and Policy University of Iowa College of Public Health Iowa City Iowa USA

**Keywords:** audio‐only telehealth, Federally Qualified Health Centers, Medicaid, mental health, utilization

## Abstract

**Objective:**

We examined how the expansion of Medicaid audio‐only coverage (MAOC) was associated with changes in mental health visit rates at Federally Qualified Health Centers (FQHCs).

**Data:**

We used publicly available FQHC‐level Uniform Data Systems (UDS) data (*N* = 9606 FQHC‐years) from 2016 to 2022. We used information from the Center for Connected Health Policy, Casetext, National Law Review, LegiScan, and other publicly available state telehealth laws and guidelines to map MAOC and other state‐level Medicaid and telehealth policy variables.

**Study Design:**

We employed a two‐way fixed effects generalized difference‐in‐differences (DiD) estimator, followed by the Callaway–Sant'Anna DiD estimator, to assess the effect of MAOC on mental health visit rates in FQHCs. The outcome was defined as mental health visit rates, and the key independent variable was MAOC policy implementation. Time‐varying state‐level covariates, including mental health provider ratio, broadband access, Medicaid and telehealth policies, as well as FQHC‐level covariates including the percentage of Medicaid‐insured and low‐income patients served, were included in the analysis. Subgroup analyses were conducted based on FQHC characteristics including rural/urban location and the presence of telemental health services (TMHS).

**Principal Findings:**

DiD analysis showed no significant effect of MAOC on mental health visit rates across FQHCs. However, subgroup analyses revealed that FQHCs without an existing TMHS experienced a 20.5% increase in visit rates (*p* < 0.05), while those with a TMHS saw a 19.73% decrease (*p* < 0.05).

**Conclusion:**

Audio‐only telehealth appears to serve as a substitute rather than a complementary modality for in‐person or video‐based mental health services in FQHCs. Providing MAOC increased mental health visit rates at FQHCs without an established telemental health service, indicating improved accessibility. Given the quality concerns surrounding audio‐only telehealth, further research is needed to validate this substitution effect and assess the quality of introducing these services as an option for FQHCs.

## Callout Box

- What is known on this topic
  ○ Medicaid reimburses mental health services for nearly 40% of low‐income Americans, positioning it as an important policy lever for expanding access to services.
  ○ Telehealth, including audio‐only (telephone‐based), plays a crucial role in improving access to mental health services, particularly among low‐income and safety‐net populations facing structural barriers.
  ○ Medicaid's audio‐only telehealth coverage for mental health services in Federally Qualified Health Centers (FQHCs) varies across states, and its impact on utilization of mental health services remains largely unknown.
- What this study found
  ○ Medicaid audio‐only coverage did not significantly change overall mental health visit rates in FQHCs across adopting and non‐adopting states.
  ○ In FQHCs without an established telemental health service, Medicaid audio‐only coverage was associated with a 20% increase in mental health visits, suggesting improved accessibility for resource‐limited clinics.
  ○ Results indicate that audio‐only services may primarily substitute for in‐person or video‐based care, raising questions about whether they expand access or simply shift the way care is delivered.

## 1 Introduction

Mental health disorders are a significant public health concern, particularly among underserved populations, where barriers to care further exacerbate their effects [1]. Low‐income individuals, rural residents, and racial and ethnic minorities experience higher rates of mental health conditions, such as depression and anxiety, often linked to social determinants such as poverty, unemployment, and limited access to healthcare [2]. These populations are also more likely to face chronic stress, trauma, and comorbid health conditions, further intensifying their mental health burden [3]. Despite the need for mental health services, access to care remains inequitable. Provider shortages, financial constraints, and geographic isolation limit treatment availability, particularly for low‐income, rural, and medically underserved populations [1]. Individuals living in rural and remote areas often travel long distances for care or go untreated [4]. Similarly, those with low socioeconomic status may struggle with out‐of‐pocket costs and insurance coverage limitations which can prevent them from accessing mental healthcare [5]. As a result, mental health disparities persist, leading to worsening symptoms, increased hospitalizations, and poorer overall health outcomes in underserved populations [6, 7].

Medicaid reimburses approximately 40% of mental health services for low‐income Americans and is a key policy lever to expand access to diagnosis and treatment of mental health disorders [8, 9]. Moreover, telehealth plays a crucial role in improving access to care for Medicaid enrollees, especially those with mental health needs [10]. Telehealth encompasses both video‐ and audio‐only modalities, each with distinct characteristics. Audio‐only telehealth refers to remote healthcare services delivered exclusively via telephone, without a visual component. This modality enables real‐time communication between patients and providers, facilitating mental health consultations, follow‐ups, and care management when video‐based interactions are not feasible due to technological or accessibility barriers. Previous studies have highlighted the potential of audio‐only (telephone‐based) telehealth to expand the use of telemental health services, particularly among low‐income and safety‐net populations [11, 12]. Audio‐only telehealth helps overcome barriers related to digital access, including lack of reliable broadband, smartphones, or computers, which are often required for video‐based services [13]. Additionally, the audio‐only option provides flexibility for individuals with limited digital literacy, privacy concerns in shared living environments, or unstable housing, thereby maintaining access to essential mental health services [14]. Considering this, many state Medicaid programs have added audio‐only coverage for mental health services to improve access to care [10, 15].

Medicaid has a separate audio‐only coverage policy for Federally Qualified Health Centers (FQHCs), which offer primary care services in areas serving low‐income, uninsured, rural, and medically underserved populations. FQHC‐specific Medicaid audio‐only coverage varies significantly from state to state, leading to state‐level disparities in the availability and reimbursement of audio‐only telehealth services [16, 17]. FQHCs provide care to approximately one‐sixth of Medicaid beneficiaries [18], and nearly 2.7 million individuals seeking mental health services [19]. Approximately 50% of all FQHC patients are covered by Medicaid/CHIP [20]. FQHCs are, therefore, pivotal in the delivery of mental health care to Medicaid‐insured and uninsured patients. Additionally, FQHCs are distinct within the safety‐net delivery system because they operate under a prospective payment system and face reimbursement and structural constraints that influence telehealth adoption differently than other primary care providers [21, 22]. As a result, several states have adopted FQHC‐specific Medicaid audio‐only coverage policies, reflecting recognition that broader Medicaid telehealth reimbursement policies may not adequately support audio‐only service delivery in FQHC settings.

Despite the growing adoption of audio‐only telehealth coverage, there is a notable lack of empirical evidence examining its impact on mental health service utilization [23]. Therefore, the objective of this study was to examine whether states that adopted Medicaid audio‐only coverage for FQHCs experienced a difference in overall utilization of mental health services compared to states that did not. This analysis provides empirical evidence on the effects of FQHC‐specific Medicaid audio‐only coverage on mental health service utilization. The findings offer insights for policymakers regarding the implications of expanding audio‐only coverage on access to care. Moreover, the results inform healthcare providers and administrators about the value of integrating audio‐only services into their telehealth services, particularly in settings such as FQHCs that serve medically underserved populations facing digital barriers.

## 2 Methods

### 2.1 Measures and Data Sources

We used publicly available FQHC‐level Uniform Data Systems (UDS) annual performance data from 2016 to 2022 (*N* = 9606 FQHC‐year observations) [24]. The key outcomes were mental health visit rates, sub‐categorized by depression and mood disorders, anxiety disorders, including post‐traumatic stress disorder (PTSD), attention deficit and disruptive behavior disorders (ADD), and other mental health disorders (Supporting Information: Appendix Table 1). According to the UDS visit guidance, a visit is defined as an encounter between a patient and a health care provider in which services are rendered and documented, and can occur in person or virtually, as long as it meets the criteria for billable services [25]. The visit rates (visits per patient) for each of these mental health conditions were calculated by dividing total number of visits for a specific mental health diagnosis by the number of patients with a specific mental health diagnosis. We interpret changes in visit rates as reflecting differences in access to and utilization of mental health services following policy adoption, rather than indicators of improved clinical care.

### 2.2 Key Independent Variable

The policy treatment of interest is “FQHC‐specific Medicaid audio‐only coverage (MAOC)” adoption status across states. We used information from the Center for Connected Health Policy, Casetext, National Law Review, LegiScan, and other publicly available state telehealth laws and guidelines to conduct policy mapping. Policy mapping involved systematically identifying, extracting, and categorizing FQHC‐specific MAOC policy information across states to create a comparable longitudinal dataset. We identified whether and when each state's Medicaid program explicitly authorized reimbursement for mental health services delivered via audio‐only modality in FQHCs. States were coded as adopters only when audio‐only reimbursement was explicitly authorized for mental health services under Medicaid in FQHCs. MAOC adoption was staggered across states, with implementation occurring in different years (Supporting Information: Appendix Table 2). Based on the year of implementation, the MAOC policy is a binary variable coded as “1” if an FQHC is located in a state that implemented audio‐only coverage policy for mental health services in that year and “0” otherwise. The adoption year of MAOC policy was defined as “treatment full effect year” if the policy was in place for more than 6 months. Once a state has adopted the policy, it continued to be treated throughout the analysis. States began adopting the MAOC policy in 2020. To examine policy effects based on the adoption year, we categorized states into three groups: 2020 adopters (10 states), 2021 adopters (4 states), and 2022 adopters (4 states) (Supporting Information: Appendix Table 2).

### 2.3 Covariates

Using UDS data, we controlled for FQHC‐level characteristics including percentage of patients with Medicaid insurance and with incomes below 200% and 100% of the federal poverty level [24]. We included state‐level variables to account for contextual factors which may influence reliance on and policy support for audio‐only services. These included (a) ratio of population to mental health providers from CMS National Provider Identification file [26], and (b) percentage of households with a broadband internet from State Health Compare [27]. To isolate the effect of FQHC‐specific MAOC, we accounted for several related state‐level Medicaid policies that evolved in a staggered manner during the study period. First, we controlled for state Medicaid expansion status, which may affect mental health service utilization through insurance coverage and demand. Second, we included two binary indicators capturing whether FQHCs were permitted to bill as distant‐site providers and originating‐site providers for telemental health services. These policies determine whether telemental health encounters are reimbursable in FQHCs and are correlated with states' broader readiness to adopt audio‐only coverage for safety‐net providers. Third, we controlled for general Medicaid audio‐only telehealth coverage that was not specific to FQHCs, which reflects states' overall orientation toward flexible telehealth audio‐only reimbursements. For these policy covariates, we used information from the Center for Connected Health Policy and other publicly available state Medicaid data sources, including state Boards of Medicine, Casetext, National Law Review, LegiScan, and state telehealth laws and guidelines to map relevant state policies.

### 2.4 Analytical Strategy

We described differences in average mental health visit rates and covariates between FQHCs in states that adopted MAOC and those that did not. We then stratified the analysis based on their year of adoption (2020, 2021, and 2022 adopters). Significance testing was performed using Kruskal‐Wallis tests for continuous variables and chi‐square tests for categorical variables.

To examine the effect of states adopting the MAOC policy on mental health visit rates in FQHCs, we compared adopting states to non‐adopting states using a two‐way fixed effects (TWFE) generalized difference‐in‐differences (DiD) estimator (Equation 1—two‐way fixed effects generalized difference‐in‐differences estimator).

(1) $$\begin{array}{c} \text{mhvisitrat}e_{\mathrm{fsy}}=\alpha +\beta {\text{MAOC}}_{\mathrm{fsy}}+X_{\mathrm{fsy}}+\delta_{f}+{ƴ}_{y}+\epsilon_{\mathrm{fsy}} \end{array}$$

$\text{mhvisitrat}e_{\mathrm{fsy}}$ represents the mental health visit rate for FQHC “*f*,” in state “*s*,” and time in years “*y*.” $\beta {\text{MAOC}}_{\mathrm{fsy}}$ is a binary treatment indicator equal to 1 in years when a state has adopted FQHC‐specific Medicaid audio‐only coverage and 0 otherwise. $X_{\mathrm{fsy}}$ is the vector for FQHC‐ and state‐level covariates. $\delta_{f}$ and ${ƴ}_{y}$ capture facility and year fixed effects, respectively. $\epsilon_{\mathrm{fsy}}$ is the error term, with standard errors clustered at the state level.

To account for the staggered nature of MAOC adoption and to provide a robustness check for our TWFE DiD estimator, we employed the Callaway–Sant'Anna DiD (CS‐DiD) estimator with doubly robust inverse probability weighting (DRIPW). This approach is well‐suited for settings with multiple treatment groups and time periods, such as our study, where states adopted MAOC in different years. The CS‐DiD estimator accounts for treatment effect heterogeneity across adoption cohorts and over time, ensuring robust and reliable DiD estimates [28]. Using this estimator, we derived the average treatment effect on the treated (ATT) for MAOC‐adopting states and the group‐time average treatment effects (GTATTs) for each adoption cohort (2020, 2021, and 2022 adopters).

(2) $$\text{mhvisitrat}e_{\mathrm{fsy}}=\alpha +\sum_{k=-K}^{K}\;{\text{βMAOC}}_{k}D_{\mathrm{fsy}}^{k}+X_{\mathrm{fsy}}+\delta_{f}+{ƴ}_{y}+\epsilon_{\mathrm{fsy}}$$

The term “$\sum_{k=-K}^{K}\;{\text{βMAOC}}_{k}D_{\mathrm{fsy}}^{k}$” in Equation (2)—CS‐DiD Estimator with GT‐ATTs represents dynamic treatment effect over time. “*k*” denotes event time relative to MAOC adoption (e.g., *k* = −1 indicates 1 year prior to adoption; *k* = 0 denotes the adoption year; *k* = 1 represents 1 year post‐adoption), and K defines the maximum number of leads and lags included in the specification. All remaining terms are defined as in Equation (1). Event‐study estimates were obtained using the CS‐DiD estimator, with all pre‐policy periods pooled as the reference (baseline) category. Event‐study plots were used to assess any pre‐policy trends prior to MAOC adoption.

We conducted two subgroup analyses: (1) urban versus rural FQHCs, and (2) FQHCs that identify mental health as a “primary telehealth” service (hereafter referred to as availability of telemental health services [TMHS]). Rural–urban classification was based on the geographic location of the health center's service delivery site, as assigned in the UDS. Stratifying by rural and urban status allows assessment of whether technology access barriers differentially influence the effects of audio‐only coverage based on geography. Restricting the sample to FQHCs with a primary TMHS allowed evaluation of MAOC effects among centers with established telehealth infrastructure. According to UDS, “primary telehealth” refers to a real‐time virtual interaction between a patient and a licensed provider, documented at the time of the encounter, and may occur via audio or video modalities. This designation does not imply that other FQHCs do not provide telehealth services; rather, it indicates that telehealth constitutes a core component of service delivery, supported by dedicated infrastructure, trained personnel, and established operational workflows.

All analyses for this study were conducted using STATA version 18.

## 3 Results

The descriptive statistics revealed differences in mental health visit rates between FQHCs in states that adopted MAOC for FQHCs and those that did not (Table 1). Among all mental health visits, the average visit rate was slightly higher for non‐adopters (3.167) compared to adopters (3.046, *p* < 0.001). Among adopters stratified by timing, 2020 adopters exhibited the highest rate (3.217), while 2021 adopters had the lowest rate (2.686, *p* < 0.001). For specific mental health conditions, significant differences were observed in rates for depression and other mood disorders, which were slightly higher for non‐adopters (3.295) than adopters (3.201, *p* < 0.01). Among adopters, 2020 adopters reported the highest rate (3.419), with 2021 adopters having the lowest rate (2.753, *p* < 0.001). Similar patterns were observed for ADDs, anxiety disorders, and other mental disorders, with significantly higher rates among non‐adopters compared to adopters. These rates were consistently highest among 2020 adopters compared to non‐adopters and later adoption years. Overall, descriptive results indicate that visit rates were generally higher among non‐adopters than adopters, however, 2020 adopters consistently demonstrated the highest utilization rates compared to non‐adopters and later adoption cohorts. To contextualize these differences in visit rates, we examined changes in both the numerator (total number of mental health visits) and the denominator (number of patients receiving mental health care) over time, which reflected the observed visit rate patterns (Supporting Information: Appendix Figures 1 and 2). Notably, among 2020 adopters, growth in visits was higher than growth in patients, resulting in a widening gap between the numerator and denominator, which indicates higher per‐patient visits.

**TABLE 1:** Descriptive Statistics for Federally Qualified Health Centers (FQHC) in States that adopted versus not‐adopted Medicaid audio‐only coverage (MAOC).

Variables	States that did not adopt and adopted MAOC for FQHCs			States that adopted MAOC for FQHCs stratified based on the timing of adoption			
	Non‐adopters (*n* = 5030)	Adopters (*n* = 4575)	Significance	2020 adopters (*n* = 2683)	2021 adopters (*n* = 1236)	2022 adopters (*n* = 656)	Significance
Outcome variables							
Mental health visits (rate^1^)							
All mental health visits	3.167	3.046	***	3.217	2.686	3.022	***
Depression and other mood disorders	3.295	3.201	**	3.419	2.753	3.151	***
Anxiety disorders, including post‐traumatic stress disorder (PTSD)	3.153	3.119	ns	3.345	2.704	2.973	***
Attention deficit and disruptive behavior disorders	3.352	3.234	**	3.297	2.97	3.459	***
Other mental disorders, excluding drug or alcohol dependence	2.949	2.666	***	2.775	2.465	2.595	***
FQHC level covariates							
Medicaid insurance (%)	41.17	46.22	***	52.77	28.52	52.76	***
FPL < 200 (%)	60.01	63.84	***	64.53	65.21	58.4	***
FPL < 100 (%)	44.04	47.40	***	47.94	48.17	43.74	***
Rural FQHCs (%)^‡^	49.36	36.06	***	30.46	45.96	40.4	***
Availability of primary telemental health service (%)^‡^	53.98	56.22	*	57.78	53.35	55.33	**
State level Covariates							
Frequent mental distress %	12.77	12.33	***	11.79	12.17	14.83	***
Mental health provider ratio	489.42	483.91	ns	388.27	679.32	506.87	***
Percentage of household with broadband	86.08	86.67	***	87.71	86.36	83	***
Presence of Medicaid expansion (%)^‡^	69.14	68.49	ns	85.76	17.07	94.82	***
Allowing FQHCs to bill as originating site providers (%)^‡^	44.99	84.48	***	98.51	70.23	53.96	***
Allowing FQHCs to bill as distance site providers (%)^‡^	59.24	64.28	***	80.13	31.47	61.28	***
Allowing audio‐only coverage for Medicaid (%)^‡^	22.37	41.32	***	42.23	39.89	40.24	***

*Note:* *p < 0.05, **p < 0.01, ***p < 0.001; ns refer to non‐significant. ^1^Rate: Number of visits by diagnosis per number of patients with diagnosis. ^‡^Refers to chi square test statistic; rest are all Kruskal–Wallis test statistics.

Other differences appeared across various covariates, highlighting differences between MAOC adopter and non‐adopter states (Table 1). FQHCs in adopter states (46.2%) served a higher percentage of Medicaid‐insured patients compared to non‐adopters (41.2%). Also, adopter states had higher percentages of patients below 200% FPL (63.84%) and 100% FPL (47.4%) than non‐adopters (60.0% and 44.0%, respectively). FQHCs in non‐adopter states (12.8%) had a slightly higher rate of frequent mental distress compared to adopters (12.3%). MAOC adopter states were more likely to implement less restrictive telehealth policies. While only 45% of FQHCs in non‐adopter states allowed FQHCs to be billed as originating site providers, this was permitted in 84.5% of adopter states. Likewise, 64.3% of adopters allowed FQHCs to bill as distance site providers, compared to 59.2% of non‐adopters. Additionally, 41.3% of adopter states provided general Medicaid coverage for audio‐only services (not for specific to FQHCs), compared to only 22.4% of non‐adopter states.

**FIGURE 1:**
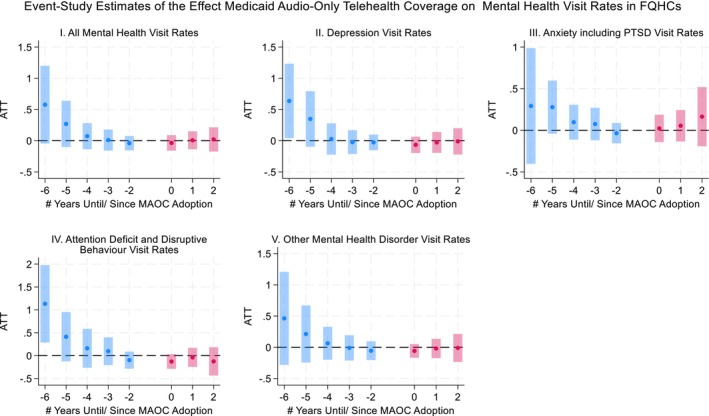
Event‐study plots using the Callaway–Sant'Anna difference‐in‐differences estimator with never adopters as the comparison group. This figure presents event‐study estimates of the effects of Medicaid audio‐only telehealth coverage (MAOC) adoption for Federally Qualified Health Centers (FQHCs) on mental health visit rates. Estimates are obtained using the Callaway–Sant'Anna difference‐in‐differences estimator for staggered treatment adoption, implemented using the long‐format aggregation approach. States that never adopted MAOC during the study period serve as the comparison group. Time 0 denotes the year of MAOC adoption in a given state. Years before and after adoption are expressed as leads (negative values) and lags (positive values) on the *x*‐axis. The year immediately prior to adoption (−1) is omitted and serves as the reference period. Points represent estimated average treatment effects, and vertical bars denote 95% confidence intervals. Blue bars indicate pre‐adoption estimates and pink bars indicate post‐adoption estimates.

**FIGURE 2:**
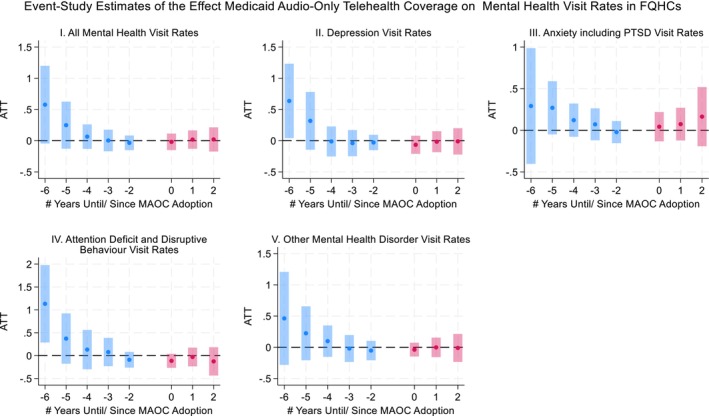
Event‐study plots using the Callaway–Sant'Anna difference‐in‐differences estimator with not yet adopters as the comparison group. This figure presents event‐study estimates of the effects of Medicaid audio‐only telehealth coverage (MAOC) adoption for Federally Qualified Health Centers (FQHCs) on mental health visit rates. Estimates are obtained using the Callaway–Sant'Anna difference‐in‐differences estimator for staggered treatment adoption, implemented using the long‐format aggregation approach. States that have not yet adopted MAOC during the study period serve as the comparison group. Time 0 denotes the year of MAOC adoption in a given state. Years before and after adoption are expressed as leads (negative values) and lags (positive values) on the *x*‐axis. The year immediately prior to adoption (−1) is omitted and serves as the reference period. Points represent estimated average treatment effects, and vertical bars denote 95% confidence intervals.

The results from both the TWFE‐DiD and CS‐DiD estimators indicated that the MAOC policy had no significant effects on mental health visit rates across all categories examined (Table 2). The TWFE estimates showed no association between MAOC adopters and mental health visit rates compared to non‐adopters. Similarly, the CS‐DiD estimates, both for all adopter groups (ATT) and stratified by adoption timing (GT‐ATTs), showed no association between MAOC and mental health visits. Results were consistent when comparing adopters to never‐adopters and not‐yet‐adopters separately. These findings are consistent with the CS‐DiD event‐study estimates (Figures 1 and 2 for ATT; Supporting Information: Appendix Figures 3–6 for GT‐ATT), which showed no statistically significant post‐adoption effects over time.

**TABLE 2:** Effect of Medicaid audio‐only telehealth coverage policy on mental health visit rates in FQHCs in adopting versus non‐adopting states.

	All mental health visit rate coefficient (SE)	Depression visit rate coefficient (SE)	Anxiety visit rate coefficient (SE)	ADD visit rate coefficient (SE)	Other mental health visit rate coefficient (SE)
TWFEs Generalized DiD Estimator					
MAOC adopters	−0.027 (0.069)	−0.038 (0.086)	−0.011 (0.079)	−0.060 (0.074)	−0.036 (0.069)
*N*	9310	9306	9304	9069	9284
Callaway–Sant' anna DiD Estimator					
Comparison group: never adopted					
ATT	−0.007 (0.069)	−0.039 (0.074)	0.068 (0.104)	−0.096 (0.093)	−0.033 (0.065)
GT‐ATTs					
2020 MAOC Adopters	−0.008 (0.092)	−0.041 (0.097)	0.056 (0.141)	−0.117 (0.117)	−0.021 (0.087)
2021 MAOC Adopters	0.022 (0.061)	−0.007 (0.070)	0.141 (0.111)	−0.027 (0.113)	−0.048 (0.057)
2022 MAOC Adopters	−0.101 (0.086)	−0.138 (0.124)	−0.073 (0.073)	−0.092 (0.078)	−0.123 (0.117)
Comparison group: not yet adopted					
ATT	0.004 (0.071)	−0.035 (0.075)	0.083 (0.107)	−0.087 (0.091)	−0.018 (0.064)
GT‐ATTs					
2020 MAOC Adopters	0.006 (0.094)	−0.030 (0.097)	0.076 (0.145)	−0.104 (0.115)	−0.002 (0.085)
2021 MAOC Adopters	0.025 (0.061)	−0.023 (0.082)	0.147 (0.111)	−0.025 (0.113)	−0.042 (0.053)
2022 MAOC Adopters	−0.101 (0.086)	−0.139 (0.124)	−0.073 (0.073)	−0.092 (0.078)	−0.123 (0.117)
*N*	9258	9254	9249	8981	9223

*Note:* Two‐way fixed effects (TWFE) and Callaway–Sant'Anna difference‐in‐differences (CS‐DiD) estimates of the effect of Medicaid audio‐only telehealth coverage on mental health visit rates in Federally Qualified Health Centers in adopting versus non‐adopting states. The table reports coefficient estimates from TWFE and CS‐DiD models estimated using ordinary least squares. Robust standard errors clustered at the state level are reported in parentheses. *N* denotes the number of FQHC‐year observations. Full model results for the TWFE generalized DiD estimator are provided in Supporting Information: Appendix Table 3. ATT denotes the average treatment effect, and GT‐ATTs denote group‐time average treatment effects by MAOC adoption year.

Event‐study analyses indicated some evidence of decreasing differential pre‐policy trends in mental health visit rates, primarily in more distant pre‐policy periods. In contrast, estimates in the periods immediately preceding MAOC adoption (−2 to −4) are centered close to zero across outcomes, supporting the absence of statistically significant pre‐trends in the years most relevant for identifying policy effects. Prior methodological work has shown that even statistically insignificant pre‐period estimates may mask underlying differential trends that affect DiD estimates. In this context, declining pre‐policy differential trends likely reflect long‐standing differences in mental health service demand across states, including structural access barriers, workforce shortages, and uneven early telehealth adoption among FQHCs, rather than from anticipation of MAOC adoption [29, 30]. As a result, pre‐existing trends are more likely to attenuate estimated effects rather than generate spurious positive findings. Accordingly, our estimates should be interpreted conservatively. Similar patterns were seen in the mental health subgroup event‐study analyses by adoption years (Supporting Information: Appendix Figures 4–7), where pre‐treatment deviations are larger and, in some cases, statistically significant in earlier periods, but diminish and center closer to zero in the years immediately preceding adoption. This pattern suggests that these subgroup estimates may also reflect longer‐run differences in underlying demand rather than policy anticipation.

In subgroup analyses, anxiety‐related visit rates among urban FQHCs in states adopting MAOC in 2022 decreased by 19.8% (*p* < 0.05), while no effects were observed for other mental health subgroups in either urban or rural FQHCs (Table 3). In the analysis stratified by the presence of a TMHS in FQHCs, significant effects of MAOC on mental health visit rates were observed among both 2020 and 2021 adopters. For FQHCs in states adopting MAOC in 2020, centers with a TMHS experienced a 19.3% decrease in overall mental health visit rates compared to those in non‐adopting states (*p* < 0.05). Depression visit rate decreased by 24.8% (*p* < 0.05), ADD visit rates decreased by 48.4% (*p* < 0.05), and visit rates for other mental health conditions decreased by 28.1% (*p* < 0.05). Conversely, in states adopting MAOC in 2021, FQHCs without a TMHS experienced a 20.5% increase in overall mental health visit rates (*p* < 0.05), including a 22.8% increase in depression visit rates (*p* < 0.05), relative to FQHCs in non‐adopting states.

**TABLE 3:** Subgroup analysis testing the effect of Medicaid audio‐only telehealth coverage policy on mental health visit rates in FQHCS by rurality, and presence of telemental health service.

Comparison group	Overall MH visit rate coefficient (SE)		Depression visit rate coefficient (SE)		Anxiety visit rate coefficient (SE)		ADD visit rate coefficient (SE)		OMH visit rate coefficient (SE)	
	NA	NYA	NA	NYA	NA	NYA	NA	NYA	NA	NYA
Rural										
ATT	−0.016 (0.101)	−0.003 (0.097)	−0.098 (0.097)	−0.086 (0.093)	0.024 (0.099)	0.042 (0.097)	0.037 (0.096)	0.041 (0.091)	0.015 (0.141)	0.032 (0.136)
GT‐ATTs										
2020 MAOC Adopters	−0.072 (0.148)	−0.050 (0.142)	−0.158 (0.136)	−0.139 (0.129)	−0.034 (0.142)	−0.005 (0.137)	0.009 (0.126)	0.016 (0.116)	−0.007 (0.215)	0.020 (0.207)
2021 MAOC Adopters	0.064 (0.082)	0.063 (0.081)	−0.014 (0.110)	−0.016 (0.109)	0.098 (0.092)	0.098 (0.092)	0.151 (0.143)	0.146 (0.143)	0.047 (0.097)	0.047 (0.094)
2022 MAOC Adopters	0.155 (0.133)	0.155 (0.133)	0.103 (0.154)	0.103 (0.154)	0.240 (0.217)	0.240 (0.217)	−0.137 (0.343)	−0.137 (0.343)	0.084 (0.123)	0.084 (0.123)
*N*	3774	3782	3772	3780	3765	3776	3610	3644	3740	3756
Urban										
ATT	−0.045 (0.186)	−0.053 (0.209)	−0.067 (0.235)	−0.083 (0.246)	0.028 (0.244)	0.014 (0.283)	−0.122 (0.205)	−0.126 (0.225)	−0.072 (0.126)	−0.065 (0.134)
GT‐ATTs										
2020 MAOC Adopters	−0.020 (0.241)	−0.030 (0.272)	−0.043 (0.307)	−0.059 (0.321)	0.037 (0.317)	0.019 (0.369)	−0.118 (0.267)	−0.124 (0.292)	−0.026 (0.157)	−0.017 (0.167)
2021 MAOC Adopters	−0.095 (0.135)	−0.098 (0.137)	−0.107 (0.150)	−0.133 (0.158)	0.059 (0.200)	0.058 (0.199)	−0.117 (0.107)	−0.112 (0.104)	−0.210 (0.124)	−0.206 (0.131)
2022 MAOC Adopters	−0.218 (0.123)	n/a	−0.255 (0.186)	n/a	−0.198* **(0.097)**	n/a	−0.193 (0.162)	n/a	−0.261 (0.178)	n/a
*N*	5211	5222	5209	5220	5208	5219	5033	5079	5194	5213
Presence of a primary telemental health service										
ATT	−0.092 (0.070)	−0.086 (0.067)	−0.140 (0.083)	−0.140 (0.081)	0.012 (0.091)	0.021 (0.089)	−0.229 (0.164)	−0.231 (0.165)	−0.176* **(0.089)**	−0.158 (0.084)
GT‐ATTs										
2020 MAOC Adopters	−0.193* **(0.091)**	−0.178* **(0.082)**	−0.248* **(0.102)**	−0.238** **(0.092)**	−0.090 (0.136)	−0.071 (0.127)	−0.484* **(0.214)**	−0.487* **(0.216)**	−0.281* **(0.130)**	−0.245* **(0.124)**
2021 MAOC Adopters	0.023 (0.105)	0.021 (0.106)	−0.017 (0.125)	−0.030 (0.130)	0.146 (0.155)	0.146 (0.155)	0.058 (0.112)	0.058 (0.112)	−0.065 (0.093)	−0.064 (0.096)
2022 MAOC Adopters	−0.075 (0.135)	−0.075 (0.135)	−0.122 (0.202)	−0.122 (0.202)	−0.031 (0.134)	−0.031 (0.134)	−0.108 (0.157)	−0.108 (0.157)	−0.125 (0.124)	−0.125 (0.124)
*N*	4313	4319	4310	4316	4307	4313	4130	4140	4295	4301
Absence of a primary telemental health service										
ATT	−0.302 (0.360)	−0.202 (0.359)	−0.494 (0.430)	−0.331 (0.408)	−0.679 (0.499)	−0.520 (0.459)	0.816 (0.869)	0.798 (0.860)	−0.035 (0.258)	−0.038 (0.252)
GT‐ATTs										
2020 MAOC Adopters	−0.539 (0.485)	−0.379 (0.507)	−0.859 (0.541)	−0.601 (0.555)	−1.126 (0.588)	−0.864 (0.585)	1.069 (1.206)	1.048 (1.194)	−0.077 (0.369)	−0.089 (0.360)
2021 MAOC Adopters	0.205* **(0.082)**	0.195* **(0.075)**	0.228* **(0.092)**	0.212* **(0.086)**	0.219 (0.118)	0.173 (0.090)	0.112 (0.304)	0.100 (0.296)	0.123 (0.124)	0.143 (0.122)
2022 MAOC Adopters	−0.248 (0.181)	−0.248 (0.181)	−0.076 (0.231)	−0.076 (0.231)	0.037 (0.240)	0.037 (0.240)	0.475 (0.280)	0.475 (0.280)	−0.289 (0.196)	−0.289 (0.196)
N	2819	3449	2817	3447	2800	3442	2781	3394	2807	3440

*Note:* Regression estimates from the Callaway–Sant'Anna difference‐in‐differences (CS‐DiD) model evaluating the effect of Medicaid Audio‐Only Telehealth Coverage on mental health visit rates in Federally Qualified Health Centers in adopting vs. non‐adopting states. The table reports coefficient estimates from the CS‐DiD model estimated using ordinary least squares. Robust standard errors clustered at the state level are reported in parentheses. *p < 0.05, **p < 0.01, ***p < 0.001. Statistically significant estimates are shown in bold. ATT denotes the average treatment effect, and GT‐ATTs denote group‐time average treatment effects. NA and NYA refer to never‐adopter and not‐yet‐adopter comparison groups, respectively. Subgroup models include all covariates specified in Equation (2).

## 4 Discussion

This study examined the impact of the MAOC policy on mental health visit rates in FQHCs, comparing centers in states that adopted the policy to those that did not. The descriptive statistics indicated early adopting states, in 2020, experienced significantly higher mental health visit rates in comparison to 2021 and 2022 adopters. One possible explanation is that MAOC adoption in 2020 coincided with the peak of the COVID‐19 pandemic, when disruptions to in‐person care created an urgent need for alternative delivery modalities, including audio‐only telehealth for mental health services [31]. These early adopting states were likely to meet this heightened demand by expanding access to audio‐only telehealth coverage. This interpretation is consistent with prior research showing that during the first 2 years of the pandemic, audio‐only visits accounted for a substantial proportion of behavioral health (2 in 5) visits in California FQHCs, before declining over time [32].

After applying TWFE and CS‐DID estimators, the observed association between MAOC and mental health service utilization diminished, and audio‐only telehealth appears to function more as a substitute. If audio‐only telehealth operated as a complementary modality, we would expect an increase in overall mental health visits due to improved access or convenience. Instead, the absence of a significant effect may imply that audio‐only telehealth might primarily be substituting for in‐person or video‐based visits, without expanding the overall volume of care. This could suggest that patients who previously accessed in‐person or video‐based care may be shifting to audio‐only services, rather than representing new patients being added to the system. Previous research suggests that telehealth serves as a ‘substitute good’ for mental health services, from both a clinical and financial perspective and that patients consider telehealth for mental health care to be substantially similar to in‐person care [33, 34]. Our findings are consistent with this substitution framework and suggest that a similar dynamic may apply to audio‐only telehealth services. If audio‐only services primarily substitute for other modalities, future research should examine whether quality of care, clinical outcomes, and patient satisfaction differ across audio‐only, video, and in‐person care [35].

These null findings are consistent with prior evidence showing that changes in Medicaid reimbursement do not uniformly increase utilization, particularly when baseline access already exists. Prior studies suggest that reimbursement expansions increase service use primarily when they ease financial or participation barriers, for example, through higher provider payment rates or improved reimbursement for specific services [36, 37]. However, such effects may be attenuated in settings like FQHCs that face capacity constraints, including limited mental health workforce availability and high clinician workloads, even when reimbursement barriers are reduced [22]. In this context, MAOC may have lowered financial barriers for audio‐only care without substantially expanding the overall volume of mental health services delivered in FQHCs.

Among urban FQHCs, a significant decline in anxiety‐related visit rates was observed in 2022 MAOC adopters. Given that this finding is limited to 1 year and one outcome, it should be interpreted cautiously, and it may reflect post‐pandemic changes in care‐seeking behavior rather than a systematic policy effect. No statistically significant effects were observed across mental health subgroups among rural FQHCs. Studies have shown that although audio‐only telehealth options may be crucial for rural populations, they still face structural barriers, including workforce shortages, digital literacy challenges, and infrastructure limitations which may hinder their ability to fully utilize audio‐only services [38]. Policy efforts need to focus on tailoring telehealth solutions to rural populations through expanding broadband availability, improving digital literacy, providing affordable and quality technology, and advocating for policies that support the sustainability of both audio‐only and video‐based telehealth services to provide equitable options for all patients.

FQHCs without an established TMHS that allowed MAOC in 2021 experienced an increase in mental health visit rates, and particularly for overall mental health and depression and mood disorders, following the policy adoption. These findings suggest that audio‐only coverage may address gaps in settings where telehealth infrastructure is limited [23]. This interpretation is consistent with prior evidence showing that Medicaid reimbursement changes increase utilization when they enable or incentivize delivery of services that were previously underprovided [39]. In this context, audio‐only coverage may have operated as a complementary modality for FQHCs lacking established telehealth infrastructure for mental health services, rather than substituting for existing care.

## 5 Policy Implications

Audio‐only telehealth may have functioned more as a substitute rather than a complement to in‐person or video‐based mental health services in FQHCs. However, among FQHCs without existing telemental health services, MAOC adoption was associated with a significant increase in mental health visits, suggesting that coverage for audio‐only services may have enhanced access and reach in under‐resourced telehealth settings. While it is important to acknowledge the importance of providing audio‐only coverage to improve access to mental health care, particularly in low‐income and safety‐net populations [23, 40], it is also important to ensure that adoption of audio‐only services in FQHCs does not compromise the quality of care or patient experience [23]. State Medicaid agencies responsible for telehealth reimbursement decisions may therefore consider audio‐only telehealth as a financially viable modality that can expand access to mental health services in FQHCs with less‐developed telemental health infrastructure, while continuing to monitor quality and patient outcomes.

Policymakers also need to consider additional measures to enhance equitable access to telemental health in FQHCs that serve rural and remote areas, including addressing digital divides, improving access to broadband and appropriate devices, strengthening internet connectivity, and supporting provider training for high‐quality virtual care across modalities [41]. Expansion of audio‐only telehealth coverage in FQHCs should be accompanied by safeguards to preserve the quality of care while leveraging its potential to improve access to mental health services in safety‐net clinics that lack the resources to support video‐based or in‐person services [23, 42]. Ongoing monitoring of clinical quality and outcomes will be important to ensure that expansion of audio‐only telehealth improves access without unintentionally widening disparities in mental health care [43].

## 6 Limitations

This study has several limitations that should be considered when interpreting the findings. First, the analysis focuses on MAOC effect on all modalities of mental health visit rates at FQHCs. A more granular examination of different visit types, such as in‐person, audio‐video, and audio‐only, would provide a better understanding of the nuanced impact of audio‐only telehealth coverage on mental health service utilization. In addition, we cannot definitively assess the substitution nature of audio‐only services because we lack granular data on in‐person or video visits. Future research is needed to further evaluate potential substitution mechanisms and their implication on overall mental health care utilization. Second, this study relies on facility‐level data, which limits the ability to capture individual patient‐level variations. Future research should analyze individual‐level data, particularly focusing on Medicaid beneficiaries receiving care in FQHCs, to better assess the effects of FQHC‐specific MAOC policies. Third, mental health visits require careful interpretation because they vary substantially in intensity and frequency. For example, visits may range from medication management to cognitive behavioral therapy or psychotherapy, which may occur monthly or more frequently. This heterogeneity complicates the use of visit counts and rates as a sole indicator of telehealth demand or access. Fourth, contemporaneous Medicaid telehealth policies, including temporary COVID‐19 related flexibilities and audio‐only coverage for non‐FQHC providers, may have shifted where Medicaid beneficiaries received mental health care, potentially diverting some demand away from FQHCs. These policies, although not specific to FQHCs or mental health services, may have attenuated any observed effects within FQHCs. Lastly, the COVID‐19 pandemic increased the burden of mental health issues, potentially driving higher utilization of services, while also serving as a catalyst for states to adopt Medicaid audio‐only coverage. These factors are challenging to control and measure, which could have introduced unaccounted confounding effects. Despite these limitations, this study adds to the limited body of research on the causal effects of audio‐only coverage policies on mental health service utilization and provides a foundation for future research.

## 7 Conclusion

This study examined the impact of the MAOC policy on mental health visit rates in FQHCs, comparing states that adopted the policy with those that did not. The overall effects of MAOC on mental health visit rates were small and non‐significant, suggesting that audio‐only telehealth might be serving more as a substitute service rather than complementary for video‐based or in‐person mental health services in FQHCs. We observed a significant increase in mental health visits at FQHCs without established telemental health services, indicating that audio‐only coverage may have improved accessibility to mental health services in under‐resourced settings. Given ongoing concerns regarding quality in audio‐only telehealth, further research should evaluate clinical outcomes, patient satisfaction, and provider perspectives on delivering mental health services through audio‐only telehealth in safety‐net settings such as FQHCs.

## Funding

The authors have nothing to report.

## Conflicts of Interest

The authors declare no conflicts of interest.

## Supporting information


**Appendix Table 1:** Diagnostic category and Applicable ICD‐10‐CN codes.
**Appendix Table 2:** States adoption status of FQHC‐specific Medicaid audio‐only coverage.
**Appendix Table 3:** Two‐way fixed effects generalized difference‐in‐differences (TWFE‐DD) regression estimates of Medicaid audio‐only telehealth coverage policy on mental health visit rates in FQHCs.
**Appendix Figure 1:** This figure presents trends in mental health service utilization in Federally Qualified Health Centers (FQHCs), stratified by Medicaid audio‐only coverage (MAOC) adopters and non‐adopters. Panels display results for all mental health conditions, depression and other mood disorders, anxiety disorders including PTSD, attention‐deficit and disruptive behavior disorders, and other mental disorders excluding substance use disorders. Across panels, solid lines show the total number of mental health visits (numerator) and dashed lines show the total number of patients with the corresponding diagnosis (denominator). These quantities represent the study's primary outcomes, visit rates defined as visits per diagnosed patient, analyzed in the main regression models. Left *y*‐axes correspond to visit counts, and right *y*‐axes correspond to patient counts.
**Appendix Figure 2:** This figure presents trends in mental health service utilization in Federally Qualified Health Centers (FQHCs), stratified by Medicaid audio‐only coverage (MAOC) adoption timing (never adopters, 2020 adopters, 2021 adopters, and 2022 adopters). Panels display results for all mental health conditions, depression and other mood disorders, anxiety disorders including PTSD, attention‐deficit and disruptive behavior disorders, and other mental disorders excluding substance use disorders. Across panels, solid lines show the total number of mental health visits (numerator) and dashed lines show the total number of patients with the corresponding diagnosis (denominator). These quantities represent the study's primary outcomes, visit rates defined as visits per diagnosed patient, analyzed in the main regression models. Left *y*‐axes correspond to visit counts, and right *y*‐axes correspond to patient counts.
**Appendix Figure 3:** Callaway–Santanna DiD estimator event study plots of the effects of Medicaid audio‐only coverage (MAOC) for Federally Qualified Health Centers (FQHCs) on mental health visit rates. Plots contain estimates and 95% confidence intervals of the effects of MAOC from the estimation of Equation (2). The time (0) was the year when MAOC was adopted in FQHCs in a given state. Years before or after relative to MAOC adoption are expressed as leads (negative values) and lags (positive values) on the *x*‐axis of the graph.
**Appendix Figure 4:** Callaway–Santanna DiD estimator event study plots of the effects of Medicaid audio‐only coverage (MAOC) for Federally Qualified Health Centers (FQHCs) on depression visit rates. Plots contain estimates and 95% confidence intervals of the effects of MAOC from the estimation of Equation (2). The time (0) was the year when MAOC was adopted in FQHCs in a given state. Years before or after relative to MAOC adoption are expressed as leads (negative values) and lags (positive values) on the *x*‐axis of the graph.
**Appendix Figure 5:** Callaway–Santanna DiD estimator event study plots of the effects of Medicaid audio‐only coverage (MAOC) for Federally Qualified Health Centers (FQHCs) on anxiety including post‐traumatic stress disorder (PTSD) visit rates. Plots contain estimates and 95% confidence intervals of the effects of MAOC from the estimation of Equation (2). The time (0) was the year when MAOC was adopted in FQHCs in a given state. Years before or after relative to MAOC adoption are expressed as leads (negative values) and lags (positive values) on the *x*‐axis of the graph.
**Appendix Figure 6:** Callaway–Santanna DiD estimator event study plots of the effects of Medicaid audio‐only coverage (MAOC) for Federally Qualified Health Centers (FQHCs) on attention deficit and disruptive behavior disorders visit rates. Plots contain estimates and 95% confidence intervals of the effects of MAOC from the estimation of Equation (2). The time (0) was the year when MAOC was adopted in FQHCs in a given state. Years before or after relative to MAOC adoption are expressed as leads (negative values) and lags (positive values) on the *x*‐axis of the graph.
**Appendix Figure 7:** Callaway–Santanna DiD estimator event study plots of the effects of Medicaid audio‐only coverage (MAOC) for Federally Qualified Health Centers (FQHCs) on attention deficit and disruptive behavior disorders visit rates. Plots contain estimates and 95% confidence intervals of the effects of MAOC from the estimation of Equation (2). The time (0) was the year when MAOC was adopted in FQHCs in a given state. Years before or after relative to MAOC adoption are expressed as leads (negative values) and lags (positive values) on the *x*‐axis of the graph.

## Data Availability

The data that support the findings of this study are openly available in Electronic Reading Room (HRSA) at https://www.hrsa.gov/foia/electronic‐reading.
