# Supplementary material for: Effect of Medicaid Audio‐Only Telehealth Coverage Policy on Mental Health Visits in Federally Qualified Health Centers
Source: Health Serv Res. 2026 Mar 26;61(2):e70107. doi: 10.1111/1475-6773.70107 (PMC13140318; doi:10.1111/1475-6773.70107)
Supplement: Supplementary file 1 — Appendix Table 1: Diagnostic category and Applicable ICD‐10‐CN codes. Appendix Table 2: States adoption status of FQHC‐specific Medicaid audio‐only coverage. Appendix Table 3: Two‐way fixed effects generalized difference‐in‐differences (TWFE‐DD) regression estimates of Medicaid audio‐only telehealth coverage policy on mental health visit rates in FQHCs. Appendix Figure 1: This figure presents trends in mental health service utilization in Federally Qualified Health Centers (FQHCs), stratified by Medicaid audio‐only coverage (MAOC) adopters and non‐adopters. Panels display results for all mental health conditions, depression and other mood disorders, anxiety disorders including PTSD, attention‐deficit and disruptive behavior disorders, and other mental disorders excluding substance use disorders. Across panels, solid lines show the total number of mental health visits (numerator) and dashed lines show the total number of patients with the corresponding diagnosis (denominator). These quantities represent the study's primary outcomes, visit rates defined as visits per diagnosed patient, analyzed in the main regression models. Left y‐axes correspond to visit counts, and right y‐axes correspond to patient counts. Appendix Figure 2: This figure presents trends in mental health service utilization in Federally Qualified Health Centers (FQHCs), stratified by Medicaid audio‐only coverage (MAOC) adoption timing (never adopters, 2020 adopters, 2021 adopters, and 2022 adopters). Panels display results for all mental health conditions, depression and other mood disorders, anxiety disorders including PTSD, attention‐deficit and disruptive behavior disorders, and other mental disorders excluding substance use disorders. Across panels, solid lines show the total number of mental health visits (numerator) and dashed lines show the total number of patients with the corresponding diagnosis (denominator). These quantities represent the study's primary outcomes, visit rates defined as v [file HESR-61-e70107-s001.docx]

## **Appendices**

**Appendix Table 1: Diagnostic category and Applicable ICD-10-CN codes**

| **Diagnostic Category** | **Applicable ICD-10-CN Code** |
| --- | --- |
| 1. Depression and other mood disorders | F30- through F39- |
| 1. Anxiety disorders, including post-traumatic stress disorder (PTSD) | F06.4, F40- through F42-, F43.0, F43.1-, F93.0 |
| 1. Attention deficit and disruptive behavior disorders | F90- through F91- |
| 1. Other mental disorders, excluding drug or alcohol dependence | F01- through F09- (exclude F06.4), F20- through F29-, F43- through F48- (exclude F43.0- and F43.1-), F50- through F99- (exclude F55-, F64-, F84.2, F90-, F91-, F93.0, F98-), O99.34-, R45.1, R45.2, R45.5, R45.6, R45.7, R45.81, R45.82, R48.0 |
| 1. All mental health disorders (all the above combined) | F30- through F39-  F06.4, F40- through F42-, F43.0, F43.1-, F93.0  F90- through F91-  F01- through F09- (exclude F06.4), F20- through F29-, F43- through F48- (exclude F43.0- and F43.1-), F50- through F99- (exclude F55-, F64-, F84.2, F90-, F91-, F93.0, F98-), O99.34-, R45.1, R45.2, R45.5, R45.6, R45.7, R45.81, R45.82, R48.0 |

**Appendix Table 2: States adoption status of FQHC-specific Medicaid Audio-only Coverage**

| ***Control: (never adopted)***  ***n=32*** | ***Treat: 2020 adopters***  ***n=10*** | ***Treat: 2021 adopters***  ***n=4*** | ***Treat: 2022 adopters***  ***n=4*** |
| --- | --- | --- | --- |
| Alabama | California | Georgia | Indiana |
| Arizona | Colorado | North Carolina | Rhode Island |
| Connecticut | Michigan | Oregon | Louisiana |
| Delaware | New York | Texas | Kentucky |
| Florida | South Carolina |  |  |
| Hawaii | South Dakota |  |  |
| Illinois | Arkansas |  |  |
| Iowa | North Dakota |  |  |
| Kansas | Virginia |  |  |
| Maine | Wisconsin |  |  |
| Maryland |  |  |  |
| Massachusetts |  |  |  |
| Minnesota |  |  |  |
| Mississippi |  |  |  |
| Missouri |  |  |  |
| Montana |  |  |  |
| Nebraska |  |  |  |
| Nevada |  |  |  |
| New Hampshire |  |  |  |
| New Jersey |  |  |  |
| New Mexico |  |  |  |
| Ohio |  |  |  |
| Oklahoma |  |  |  |
| Pennsylvania |  |  |  |
| Tennessee |  |  |  |
| Utah |  |  |  |
| Vermont |  |  |  |
| West Virginia |  |  |  |
| Wyoming |  |  |  |
| Idaho |  |  |  |
| Alaska |  |  |  |
| Washington |  |  |  |

**Appendix Table 3: Two-way fixed Effects Generalized Difference-in-differences (TWFE-DD) regression estimates of Medicaid audio-only telehealth coverage policy on mental health visit rates in FQHCs**

|  | All Mental health visit rate  Coefficient  (SE) | Depression visit rate  Coefficient  (SE) | Anxiety visit rate  Coefficient  (SE) | ADD visit rate  Coefficient  (SE) | Other mental health visit rate  Coefficient  (SE) |
| --- | --- | --- | --- | --- | --- |
| MAOC for FQHCs | -0.027 | -0.038 | -0.011 | -0.060 | -0.036 |
|  | (0.069) | (0.086) | (0.079) | (0.074) | (0.069) |
| Year effects (ref:2016) |  |  |  |  |  |
| 2017 | 0.164*** | 0.141** | 0.232*** | 0.131** | 0.089* |
|  | (0.036) | (0.051) | (0.059) | (0.046) | (0.044) |
| 2018 | 0.274*** | 0.235** | 0.347*** | 0.224** | 0.218** |
|  | (0.064) | (0.084) | (0.086) | (0.064) | (0.070) |
| 2019 | 0.444*** | 0.422*** | 0.557*** | 0.405*** | 0.324** |
|  | (0.079) | (0.101) | (0.113) | (0.059) | (0.093) |
| 2020 | 0.849*** | 0.929*** | 1.035*** | 0.671*** | 0.554*** |
|  | (0.117) | (0.149) | (0.180) | (0.090) | (0.126) |
| 2021 | 0.993*** | 1.089*** | 1.230*** | 0.803*** | 0.657*** |
|  | (0.145) | (0.181) | (0.224) | (0.104) | (0.155) |
| 2022 | 0.980*** | 1.079*** | 1.247*** | 0.801*** | 0.586** |
|  | (0.160) | (0.195) | (0.256) | (0.120) | (0.167) |
| Medicaid | 0.003 | 0.004 | 0.003 | 0.003 | 0.001 |
|  | (0.003) | (0.004) | (0.004) | (0.003) | (0.003) |
| FPL below 200 | 0.001 | 0.000 | 0.002 | 0.001 | 0.001 |
|  | (0.002) | (0.002) | (0.002) | (0.003) | (0.002) |
| FPL below 100 | 0.003 | 0.003 | 0.001 | 0.001 | 0.002 |
|  | (0.002) | (0.003) | (0.002) | (0.003) | (0.003) |
| ^†^Mental health provider ratio | 0.001* | 0.001 | 0.001 | 0.001** | 0.001 |
|  | (0.000) | (0.000) | (0.000) | (0.000) | (0.001) |
| ^†^Broadband availability | -0.029* | -0.027 | -0.041 | -0.009 | -0.019 |
|  | (0.012) | (0.013) | (0.023) | (0.014) | (0.013) |
| ^†^Medicaid Expansion | -0.077 | -0.098 | -0.150 | -0.045 | -0.052 |
|  | (0.079) | (0.088) | (0.090) | (0.095) | (0.094) |
| ^†^Originating site restriction | 0.278 | 0.347 | 0.315 | 0.119 | 0.184 |
|  | (0.154) | (0.205) | (0.186) | (0.118) | (0.097) |
| ^†^Distant site restriction | 0.030 | 0.007 | -0.018 | 0.055 | 0.077 |
|  | (0.064) | (0.073) | (0.072) | (0.067) | (0.070) |
| ^†^Medicaid audio only coverage | 0.066 | 0.041 | 0.092 | 0.009 | 0.084 |
|  | (0.063) | (0.069) | (0.061) | (0.077) | (0.082) |
| _cons | 4.155*** | 4.190*** | 5.188* | 2.692* | 3.317* |
|  | (1.139) | (1.178) | (2.039) | (1.336) | (1.314) |
| *N* | 9320 | 9316 | 9313 | 9077 | 9294 |

* p < 0.05, ** p < 0.01, *** p < 0.001. The table contains of coefficient estimates derived from the TWFs DiD using ordinary least squares regression. Robust standard errors clustered at state level in parentheses. N refers to the sample size

*
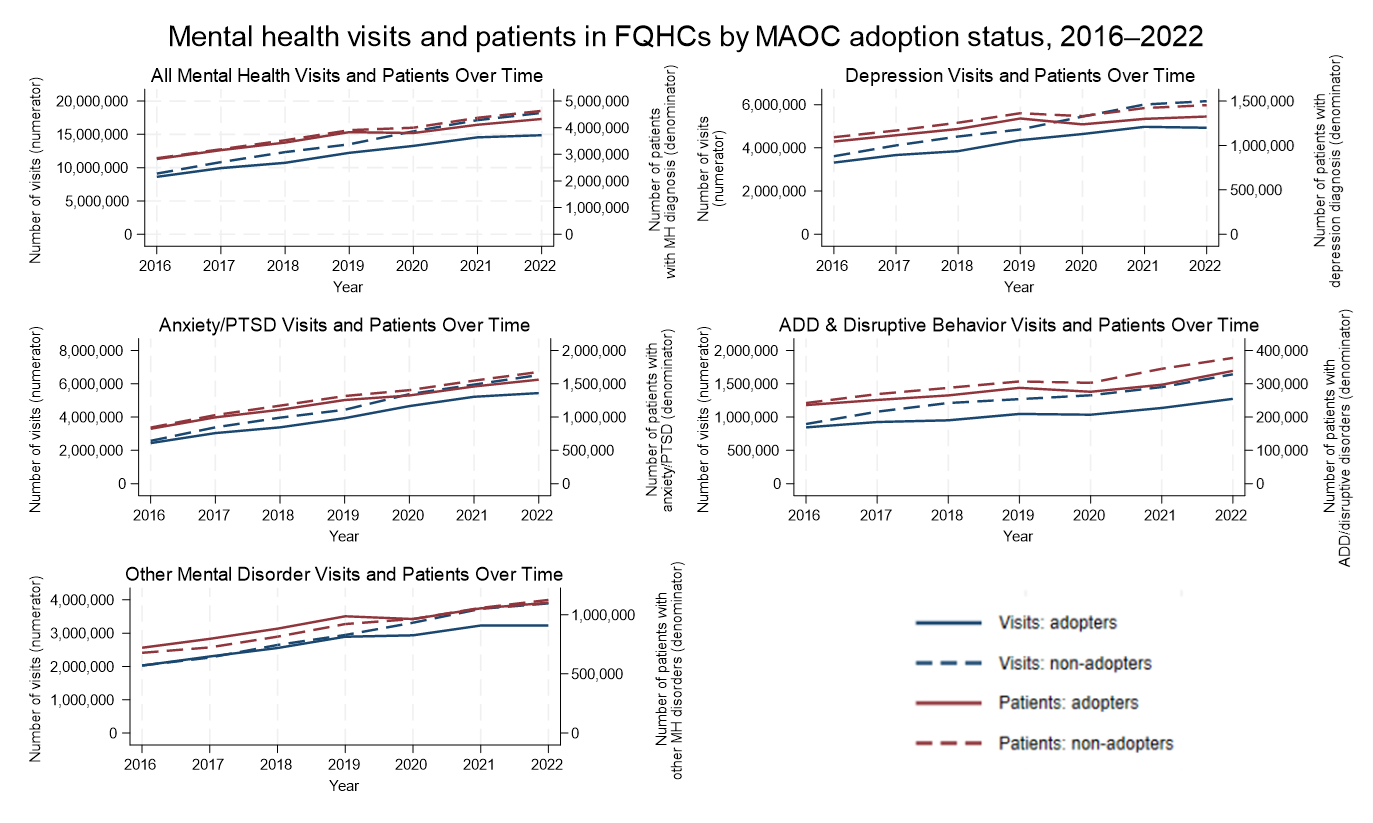
*

Appendix Figure 1: This figure presents trends in mental health service utilization in Federally Qualified Health Centers (FQHCs), stratified by Medicaid audio-only coverage (MAOC) adopters and non-adopters. Panels display results for all mental health conditions, depression and other mood disorders, anxiety disorders including PTSD, attention-deficit and disruptive behavior disorders, and other mental disorders excluding substance use disorders. Across panels, solid lines show the total number of mental health visits (numerator) and dashed lines show the total number of patients with the corresponding diagnosis (denominator). These quantities represent the study’s primary outcomes, visit rates defined as visits per diagnosed patient, analyzed in the main regression models. Left y-axes correspond to visit counts, and right y-axes correspond to patient counts.

*
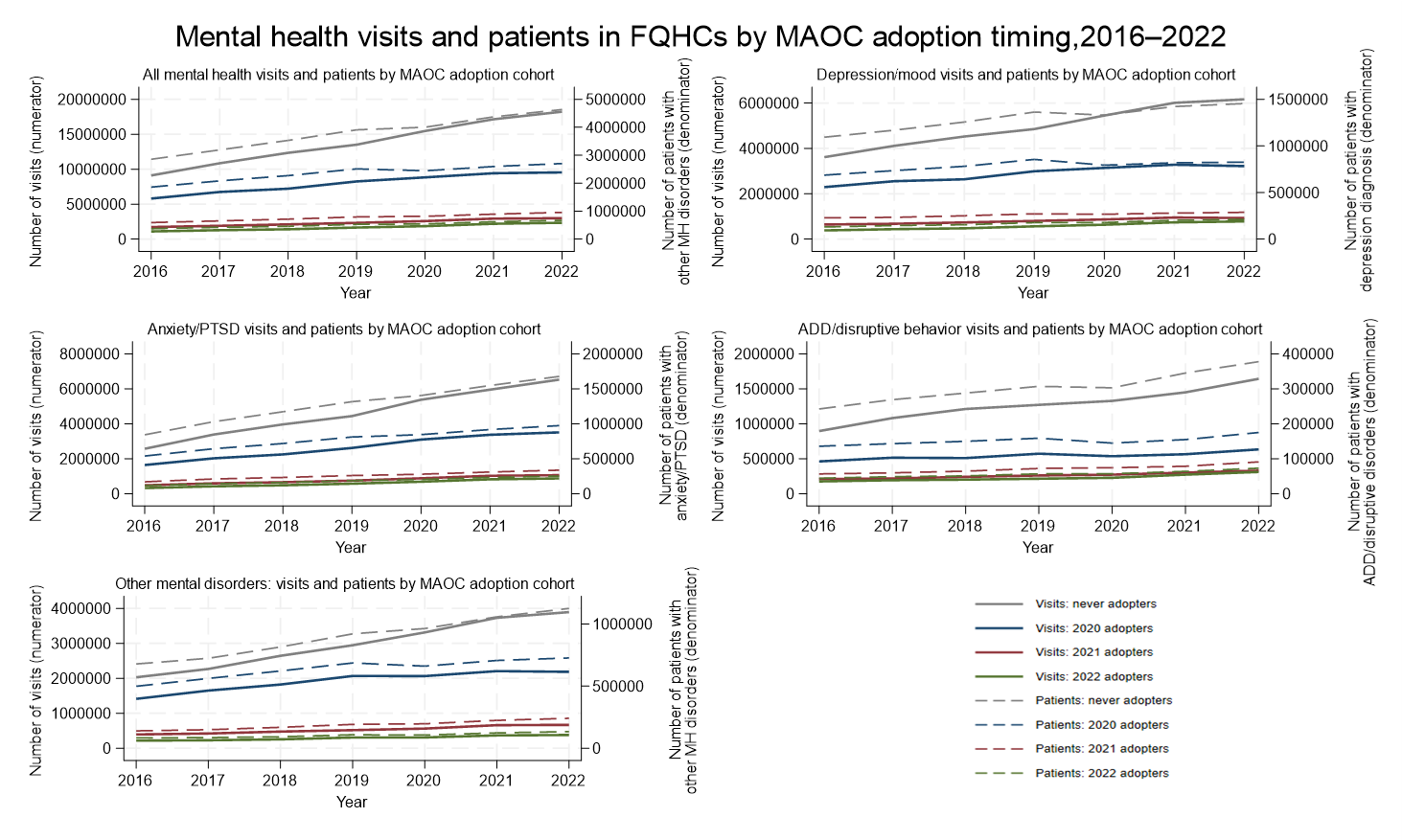
*

Appendix Figure 2: This figure presents trends in mental health service utilization in Federally Qualified Health Centers (FQHCs), stratified by Medicaid audio-only coverage (MAOC) adoption timing (never adopters, 2020 adopters, 2021 adopters, and 2022 adopters). Panels display results for all mental health conditions, depression and other mood disorders, anxiety disorders including PTSD, attention-deficit and disruptive behavior disorders, and other mental disorders excluding substance use disorders. Across panels, solid lines show the total number of mental health visits (numerator) and dashed lines show the total number of patients with the corresponding diagnosis (denominator). These quantities represent the study’s primary outcomes, visit rates defined as visits per diagnosed patient, analyzed in the main regression models. Left y-axes correspond to visit counts, and right y-axes correspond to patient counts.

Appendix Figure 3: Callaway-Santanna DiD estimator event study plots of the effects of Medicaid audio-only coverage (MAOC) for Federally Qualified Health Centers (FQHCs) on mental health visit rates. Plots contain estimates and 95% confidence intervals of the effects of MAOC from the estimation of Equation [2](https://onlinelibrary.wiley.com/doi/full/10.1111/1475-6773.14125#hesr14125-disp-0002). The time (0) was the year when MAOC was adopted in FQHCs in a given state. Years before or after relative to MAOC adoption are expressed as leads (negative values) and lags (positive values) on the x-axis of the graph.

Appendix Figure 4: Callaway-Santanna DiD estimator event study plots of the effects of Medicaid audio-only coverage (MAOC) for Federally Qualified Health Centers (FQHCs) on depression visit rates. Plots contain estimates and 95% confidence intervals of the effects of MAOC from the estimation of Equation [2](https://onlinelibrary.wiley.com/doi/full/10.1111/1475-6773.14125#hesr14125-disp-0002). The time (0) was the year when MAOC was adopted in FQHCs in a given state. Years before or after relative to MAOC adoption are expressed as leads (negative values) and lags (positive values) on the x-axis of the graph.

Appendix Figure 5: Callaway-Santanna DiD estimator event study plots of the effects of Medicaid audio-only coverage (MAOC) for Federally Qualified Health Centers (FQHCs) on anxiety including post-traumatic stress disorder (PTSD) visit rates. Plots contain estimates and 95% confidence intervals of the effects of MAOC from the estimation of Equation [2](https://onlinelibrary.wiley.com/doi/full/10.1111/1475-6773.14125#hesr14125-disp-0002). The time (0) was the year when MAOC was adopted in FQHCs in a given state. Years before or after relative to MAOC adoption are expressed as leads (negative values) and lags (positive values) on the x-axis of the graph.

Appendix Figure 6: Callaway-Santanna DiD estimator event study plots of the effects of Medicaid audio-only coverage (MAOC) for Federally Qualified Health Centers (FQHCs) on attention deficit and disruptive behaviour disorders visit rates. Plots contain estimates and 95% confidence intervals of the effects of MAOC from the estimation of Equation [2](https://onlinelibrary.wiley.com/doi/full/10.1111/1475-6773.14125#hesr14125-disp-0002). The time (0) was the year when MAOC was adopted in FQHCs in a given state. Years before or after relative to MAOC adoption are expressed as leads (negative values) and lags (positive values) on the x-axis of the graph.

Appendix Figure 7: Callaway-Santanna DiD estimator event study plots of the effects of Medicaid audio-only coverage (MAOC) for Federally Qualified Health Centers (FQHCs) on attention deficit and disruptive behaviour disorders visit rates. Plots contain estimates and 95% confidence intervals of the effects of MAOC from the estimation of Equation [2](https://onlinelibrary.wiley.com/doi/full/10.1111/1475-6773.14125#hesr14125-disp-0002). The time (0) was the year when MAOC was adopted in FQHCs in a given state. Years before or after relative to MAOC adoption are expressed as leads (negative values) and lags (positive values) on the x-axis of the graph.
